# Supplementary material for: Sex-Specific Metabolic Pathways Were Associated with Alzheimer’s Disease (AD) Endophenotypes in the European Medical Information Framework for AD Multimodal Biomarker Discovery Cohort
Source: Biomedicines. 2021 Nov 3;9(11):1610. doi: 10.3390/biomedicines9111610 (PMC8615383; doi:10.3390/biomedicines9111610)
Supplement: Supplementary file 1 [file biomedicines-09-01610-s001.zip › biomedicines-1428756-Supplementary.pdf]

Supplementary document for:

Sex-specific metabolic pathways associate with Alzheimer's Disease (AD) endophenotypes in the European Medical Information Framework for AD Multimodal Biomarker Discovery cohort

Jin Xu, Rebecca Green, Min Kim, Jodie Lord, Amera Ebshiana, Sarah Westwood, Alison Baird, Alejo Nevado-Holgado, Liu Shi. Abdul Hye, Stuart G Snowden, Isabelle Bos, Stephanie J B Vos, Rik Vandenberghe, Charlotte E Teunissen, Mara Ten Kate, Philip Scheltens, Silvy Gabel, Karen Meersmans, Olivier Blin, Jill Richardson, Ellen Elisa De Roeck, Sebastiaan Engelborghs, Kristel Sleegers, Régis Bordet, Lorena Rami, Petronella Kettunen, Magda Tsolaki, Frans R J Verhey, Daniel Alcolea, Alberto Lleó, Gwendoline Peyratout, Mikel Tainta, Peter Johannsen, Yvonne Freund-Levi, Lutz Frölich, Valerija Dobricic, Giovanni B Frisoni, José Luis Molinuevo, Anders Wallin, Julius Popp, Pablo Martinez-Lage, Lars Bertram, Kaj Blennow, Henrik Zetterberg, Johannes Streffer, Pieter Jelle Visser, Simon Lovestone, Petroula Proitsi*, Cristina Legido-Quigley*

Corresponding authors:

Cristina Legido-Quigley cristina.legido.quigley@regionh.dk

Petroula Proitsi petroula.proitsi@kcl.ac.uk

Supplementary methods and results

Pathway enrichment analysis

The hypergeometric test was performed to identify pathways that are enriched in females and males in collections of AD endophenotypes (diagnosis, CSF markers, cognition, and brain MRI) for metabolites showing associations at the nominal significance level in sex stratified analyses (1)(2). P-values were adjusted for false discovery rate (FDR) at a corrected P<0.05.

Each known metabolite was classified into one of the following eight "super-pathways": 'Amino acid', 'Carbohydrate', 'Cofactors and vitamins' 'Energy', 'Lipid', 'Nucleotide', 'Peptide', and 'Xenobiotics' by *Metabolon* based on the Kyoto Encyclopaedia of Genes and Genomes (KEGG) database. Each super-pathway was further subdivided into two or more "sub-pathways". The original 60 sub-pathways pre-defined by *Metabolon* for all 665 metabolites were employed as reference. Over-representation analyses using the hypergeometric test were performed for the metabolites associated in the sex-stratified analyses with the collections of endophenotypes in females and males respectively. Those containing less than 5 metabolites were excluded, resulting in 34 sub-pathways (10 sub-pathways for diagnosis, 10 for CSF biomarkers, 27 for cognition outcomes and 16 for brain MRI measurements) in the female dataset and 39 sub-pathways (10 sub-pathways for diagnosis, 10 for CSF biomarkers, 26 for cognition outcomes and 18 for brain MRI measurements) in the male dataset. It was revealed that 'vitamin A metabolism' (q = 5.7e-04) and 'tryptophan metabolism' sub-pathways (q = 2.4e-02) were enriched for diagnosis in the female group. Three additional sub-pathways were associated with cognition outcomes and four sub-pathways with brain MRI measurements in females (Supplementary Table S2), including 'acyl carnitine fatty acid metabolism' (q = 8.4e-04), 'monohydroxy fatty acid' (q = 1.7e-02), 'leucine, isoleucine and valine metabolism' (q = 4.1e-02), 'sphingolipid metabolism' (q = 3.4e-

02), ‘phosphatidylinositol (PI)’ (q = 1.9e-02), ‘primary bile acid metabolism’ (q = 3.4e-02), and ‘plasmalogen’ (q = 3.4e-04). Ceramides were enriched for both cognition (q = 6.6e-04) and brain MRI measurements (q = 1.7e-04) in males.

The hypergeometric analysis highlighted nine sub-pathways in females and one in males. In females, both vitamin A metabolism (3) and tryptophan metabolism pathways (4) were correlated with diagnosis. The other important sub-pathways in females included acyl carnitine metabolism, phosphatidylinositol, as well as leucine, isoleucine and valine (5) which have been reported as combating oxidative stress, AD pathology or cognition. It has been demonstrated in other studies that the metabolism of some acyl-carnitines is finely connected among different diagnostic groups, as well as with age and sex (6). In males, the ceramides sub-pathway, which has been widely reported to play a role in AD and other neurodegenerative disorders was enriched (7).

Association analyses including *APOE ε4* as stratification variable

Studies have reported that *APOE ε4* genotype may exert AD risk in a sex-dependent manner, which has a greater influence on female *APOE ε4* carrier (8–11).To investigate potential influence of *APOE ε4* status on the metabolomic level, we also performed association analyses with all 15 AD endophenotypes from four categories, now stratified by *APOE ε4* status and adjusted for sex. We found that 118 metabolites showed asscociation with AD endophenotypes at nominal significance level in the *APOE ε4* carrier stratum, while 116 metabolites were predominant in the non-carrier stratum (supplementary table S2). Similarly, there were 22 and 18 metabolites associated with AD endophenotypes in *APOE ε4* + and *APOE ε4* – stratum at Bonferroni significance threshold ($P < 8.17e-04$), respectively.

After comparing the metabolites identified in either sex group and *APOE* ε4 carrier stratification analyses (Supplementary Table S1 and Table S2). It was noticed that one metabolite, 2-Hydroxyisocaproic acid, showed association with immediate memory at nominal level in both female and *APOE* ε4 carrier strata (Supplementary Table S3 & Figure S15F). Additionally, the overlaps of metabolites between male statum and *APOE* ε4 carrier stratum at nominal level include LPC (18:2) and methyl glucopyranoside (alpha + beta) (associated with AD), cystathionine (associated with attention), Cer(d18:1/16:0) and GlcCer(d18:1/16:0) (associated with visuoconstruction) (Supplementary Table S3 & Figure S15A-E). Finally, when we investigated further for the cross-over overlapping metabolites that showed association with the the same endophenotype from sex strata at Bonferroni threshold and *APOE* ε4 strata at nominal level, as well as those from *APOE* ε4 starta at Bonferroni threshold and sex starta at nominal level, LPC (18:2) was found to be the only metabolite that associated with AD in males (P = 1.11e-03) and *APOE* ε4 carriers (P = 3.92e-04).

1. Michonneau D, Latis E, Curis E, Dubouchet L, Ramamoorthy S, Ingram B, et al. Metabolomics analysis of human acute graft-versus-host disease reveals changes in host and microbiota-derived metabolites. Nat Commun. 2019 13;10(1):5695.
2. Lewis KN, Rubinstein ND, Buffenstein R. A window into extreme longevity; the circulating metabolomic signature of the naked mole-rat, a mammal that shows negligible senescence. GeroScience. 2018;40(2):105–21.
3. Ono K, Yamada M. Vitamin A and Alzheimer's disease. Geriatr Gerontol Int. 2012 Apr;12(2):180–8.
4. Lovelace MD, Varney B, Sundaram G, Lennon MJ, Lim CK, Jacobs K, et al. Recent evidence for an expanded role of the kynurenine pathway of tryptophan metabolism in neurological diseases. Neuropharmacology. 2017 Jan 1;112:373–88.
5. Tynkkynen J, Chouraki V, van der Lee SJ, Hernesniemi J, Yang Q, Li S, et al. Association of branched-chain amino acids and other circulating metabolites with risk of incident dementia and Alzheimer's disease: A prospective study in eight cohorts. Alzheimers Dement J Alzheimers Assoc. 2018;14(6):723–33.

6. Serum Levels of Acyl-Carnitines along the Continuum from Normal to Alzheimer's Dementia [Internet]. [cited 2020 Sep 17]. Available from: https://journals.plos.org/plosone/article?id=10.1371/journal.pone.0155694

7. Kim M, Nevado-Holgado A, Whiley L, Snowden SG, Soininen H, Kloszewska I, et al. Association between Plasma Ceramides and Phosphatidylcholines and Hippocampal Brain Volume in Late Onset Alzheimer's Disease. J Alzheimers Dis. 2017;60(3):809–17.

8. Farrer LA, Cupples LA, Haines JL, Hyman B, Kukull WA, Mayeux R, et al. Effects of age, sex, and ethnicity on the association between apolipoprotein E genotype and Alzheimer disease. A meta-analysis. APOE and Alzheimer Disease Meta Analysis Consortium. JAMA. 1997 Oct 22;278(16):1349–56.

9. Altmann A, Tian L, Henderson VW, Greicius MD, Alzheimer's Disease Neuroimaging Initiative Investigators. Sex modifies the APOE-related risk of developing Alzheimer disease. Ann Neurol. 2014 Apr;75(4):563–73.

10. Mortensen EL, Høgh P. A gender difference in the association between APOE genotype and age-related cognitive decline. Neurology. 2001 Jul 10;57(1):89–95.

11. Neu SC, Pa J, Kukull W, Beekly D, Kuzma A, Gangadharan P, et al. Apolipoprotein E Genotype and Sex Risk Factors for Alzheimer Disease: A Meta-analysis. JAMA Neurol. 2017 Oct 1;74(10):1178–89.

Supplementary Tables

Table S1. Number of metabolites showed association with each AD endophenotypes in the whole cohort and each sex stratum.

Category	*Outcomes*	*All(*sex) p<0.05*		*Female p<0.05*		*Male p<0.05*		*Female p<8.17e-04*	*Male p<8.17e-04*
Diagnosis	AD/CTL	30		15		13		2	1
CSF markers	P-tau	11	(38)	5	(15)	5	(13)		
	T-tau	2		6		1			
	Amyloid-beta	26		6		7			
Cognition	Attention	13	(152)	7	(66)	7	(60)		
	Executive function	48		16		7			
	Language	27		6		19			
	Memory Delay	27		18		8			
	Memory Immediate	26		19		6			
	Visuoconstruction	34		10		17			
Brain MRI	Hippocampal Left	23	(79)	8	(35)	10	(33)		
	Hippocampal Right	26		6		12			
	Hippocampal Sum	22		6		10			
	Cortical thickness WB	28		9		8			
	Cortical thickness AD	43		21		14			
Total		**230**		**116**		**100**			

WB, whole brain; AD, Alzheimer's disease; CTL, control.

Table S2. Number of metabolites showed association with AD endophenotypes in the whole cohort and each *APOE* ε4 +/- stratum.

Category	*Outcomes*	*All(*APOE ε4 +/-) p<0.05*	*APOE ε4 + p<0.05*		*APOE ε4 - p<0.05*		*APOE ε4 + p<8.17e-04*		*APOE ε4 - p<8.17e-04*	
Diagnosis	AD/CTL	42	23		13		7		1	
CSF markers	P-tau	65	25		34		1		8	
	T-tau	66	24	(40)	37	(60)	-	(2)	10	(16)
	Amyloid-beta	31	6		12		1		3	
Cognition	Attention	35	16		12		1		2	
	Executive function	21	8		8		-		1	
	Language	9	3	(62)	3	(41)	2	(8)	1	(5)
	Memory Delay	44	16		8		3		-	
	Memory Immediate	32	13		10		2		2	
	Visuoconstruction	43	11		4		-		-	
Brain MRI	Hippocampal Left	18	8		4		2		-	
	Hippocampal Right	12	3		5		1		-	
	Hippocampal Sum	15	7	(15)	5	(14)	2	(2)	1	
	Cortical thickness WB	14	5		3		-		-	
	Cortical thickness AD	17	4		5		-		-	
Total			**118**		**116**		**18**		**22**	

WB, whole brain; AD, Alzheimer's disease; CTL, control.

Table S3. **Overlap of of metabolites showed association with AD endophenotypes in the stratification analyses between female/male group and *APOE* ε4 + group at nominal significance levels (*p* < 0.05).**

Sex	AD endophenotypes	*APOE ε*4 + group
Male	Diagnosis	LPC (18:2)
		methyl glucopyranoside (alpha + beta)
	Attention	Cystathionine
	Visuoconstruction	Cer(d18:1/16:0)
		GlcCer(d18:1/16:0)
Female	Memory Immediate	2-Hydroxyisocaproic acid

Table S4. **The associations of the ten sub-pathways with four collections of AD endophenotypes in both sex strata.**

		Pathways	P-value	q-value
Female	Diagnosis	Vitamin A metabolism	5.73e-05	5.7e-04
		Tryptophan metabolism	4.71e-03	2.4e-02
	Cognition	Acyl Carnitine metabolism	3.10e-05	8.4e-04
		Monohydroxy fatty acid	1.26e-03	1.7e-02
		Leucine, isoleucine and valine metabolism	4.58e-03	4.1e-02
	MRI	Phosphatidylinositol metabolism	1.17e-03	1.9e-02
		Sphingolipid metabolism	4.28e-03	3.4e-02
		Primary bile acid metabolism	6.29e-03	3.4e-02
		Plasmalogen	8.40e-03	3.4e-02
Male	Cognition	Ceramides	2.54e-05	6.6e-04
	MRI	Ceramides	9.60e-06	1.7e-04

Table S5. Bootstrapping results of standard error (SE) and 95% confident interval for AUCs from ROC curves.

	AUC			SE			95% confidence interval		
	Female	Male	All	Female	Male	All	Female	Male	All
VMA + Tryptophan Betaine	0.834	0.639	0.742	0.029	0.046	0.027	0.777 – 0.889	0.546 – 0.733	0.687 – 0.795
Kynurenate	0.589	0.679	0.623	0.043	0.046	0.031	0.505 – 0.672	0.593 – 0.765	0.562 – 0.682
P-tau	0.748	0.783	0.762	0.037	0.042	0.027	0.672 – 0.816	0.697 – 0.862	0.707 – 0.813
T-tau	0.824	0.877	0.845	0.031	0.035	0.023	0.756 – 0.881	0.805 – 0.942	0.798 – 0.887
Aβ	0.839	0.803	0.824	0.029	0.035	0.022	0.775 – 0.892	0.734 – 0.865	0.781 – 0.866
CSF markers	0.885	0.908	0.894	0.025	0.026	0.018	0.836 – 0.93	0.852 – 0.954	0.858 – 0.926
Covariates	0.826	0.77	0.801	0.031	0.042	0.024	0.763 – 0.886	0.68 – 0.848	0.752 – 0.846
CSF markers + covariates	0.92	0.932	0.921	0.019	0.019	0.014	0.881 – 0.954	0.89 – 0.965	0.893 – 0.948
VMA + Tryptophan Betaine + covariates	0.887	-	0.83	0.024	-	0.023	0.836 – 0.931	-	0.784 – 0.874
Kynurenate + covariates	-	0.815	0.813	-	0.035	0.023	-	0.742 – 0.878	0.766 – 0.854
VMA + Tryptophan betaine + CSF markers + covariates	0.955	-	0.939	0.012	-	0.012	0.93 – 0.978	-	0.914 – 0.962
Kynurenate + CSF markers + covariates	-	0.932	0.921	-	0.02	0.014	-	0.888 – 0.966	0.893 – 0.948

Covariates include age at sampling, education years, sampling batch and *APOE ε4* status; CSF makers include p-tau, t-tau and Aβ; AUC, area under curve; ROC, receiver operating characteristic; VMA, vanillymadelate, Aβ, Amyloid beta.

Table S6. List of collaborators in European Medical Information Framework (EMIF) consortium.

Organisations	Partner Type	Country
Aarhus Universitetshospital	Academia	Denmark
Agenzia Regionale di Sanità	Academia	Italy
Alzheimer Europe	Patient Organisation	Luxembourg
Amgen	EFPIA	UK
Boehringer Ingelheim International GmbH	EFPIA	Germany
Cambridge Cognition Ltd.	SME	UK
Concentris Research Management GmbH	SME	Germany
Custodix NV	SME	Belgium
Electrophoretics Ltd.	SME	UK
Erasmus Universitair Medisch Centrum Rotterdam	Academia	The Netherlands
European Institute for Health Records	Academia	Belgium
European Molecular Biology Laboratory	Academia	Germany
F. Hoffmann-La Roche AG	EFPIA	Switzerland
Fondazione PENTA—for the Treatment and Care of Children with HIV-ONLUS	Academia	Italy
Genomedics S.R.L.	Academia	Italy
GlaxoSmithKline Research and Development Ltd.	EFPIA	UK
Göteborgs Universitet	Academia	Sweden
Helsingin Yliopisto	Academia	Finland
IDIAP	Academia	Spain
Institut National de la Santé et de la Recherche Médicale	Academia	France
Itä-Suomen Yliopisto	Academia	Finland
Janssen Pharmaceutica NV	EFPIA	Belgium
Karolinska Institutet	Academia	Sweden
King's College London	Academia	UK
Leibniz Institute for Prevention, Research and Epidemiology—BIPS GmbH	Academia	Germany
Maastricht University	Academia	The Netherlands
MAAT France	SME	France
Merck	EFPIA	Germany
Novo Nordisk A/S	EFPIA	Denmark
Pedianet	SME	Italy
Pfizer	EFPIA	UK
Pharmo Cooperatie UA	SME	The Netherlands
Provincia Lombardo-Veneta—Ordine Ospedaliero di San Giovanni di Dio—Fatebenefratelli	Academia	Italy
Servier	EFPIA	France
Synapse Research Management Partners S.L.	SME	Spain
Tartu Ulikool	Academia	Estonia
Teknologian tutkimuskeskus VTT	Academia	Finland
UCB Pharma SA	EFPIA	Belgium
Universidade de Aveiro	Academia	Portugal
Universitat Pompeu Fabra	Academia	Spain
Universiteit Antwerpen	Academia	Belgium

University College London	Academia	UK
University of Copenhagen	Academia	Denmark
University of Exeter	Academia	UK
University of Glasgow	Academia	UK
University of Leicester	Academia	UK
University of Manchester	Academia	UK
University of Oxford	Academia	UK
University Pierre et Marie Curie	Academia	France
Università degli Studi di Pisa	Academia	Italy
Universität Leipzig	Academia	Germany
Universität zu Lübeck (University of Lübeck)	Academia	Germany
Universitätsklinikum Erlangen, Anstalt des Öffentlichen Rechts	Academia	Germany
Vestische Kinder—und Jugendklinik Datteln	Academia	Germany
VIB	Academia	Belgium
VU University Medical Center	Academia	The Netherlands

Supplementary Figures

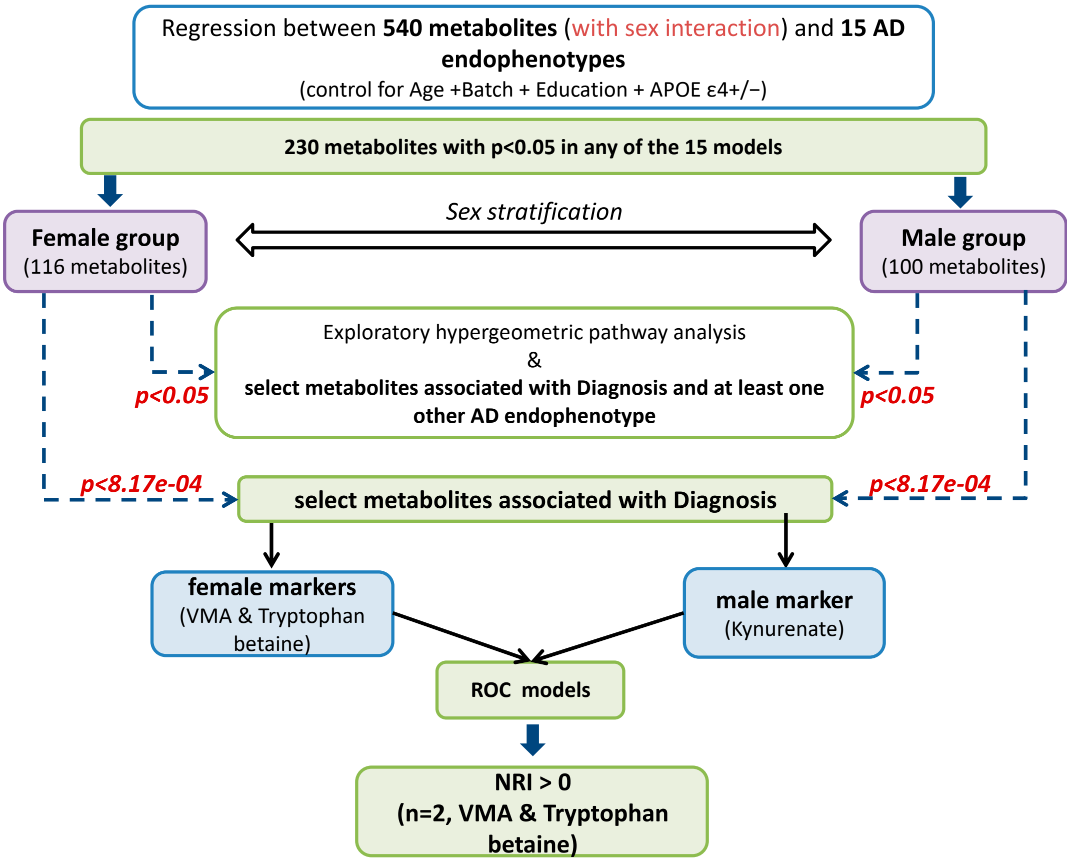

Figure S1. Study workflow. The associations between 540 plasma metabolites with a sex interaction term and 15 AD endophenotypes were investigated using linear/logistic regression. These models were repeated for 230 significant metabolites that showed interactions with sex. Metabolites that i) passed the P<0.05 threshold and correlated with any of 15 AD endophenotypes were further employed for exploratory hypergeometric pathway analysis in four categories of AD endophenotypes; ii) passed the P<0.05 threshold and correlated with both diagnosis as well as any other endophenotype were investigated; iii) passed the p<8.17e-04 threshold in each sex stratum and showed association with diagnosis (AD/CTL) were selected for regession models followed by receiver operating characteristic (ROC) curves to test their prediction abilities in AD, which was further validated using net reclassification index (NRI).

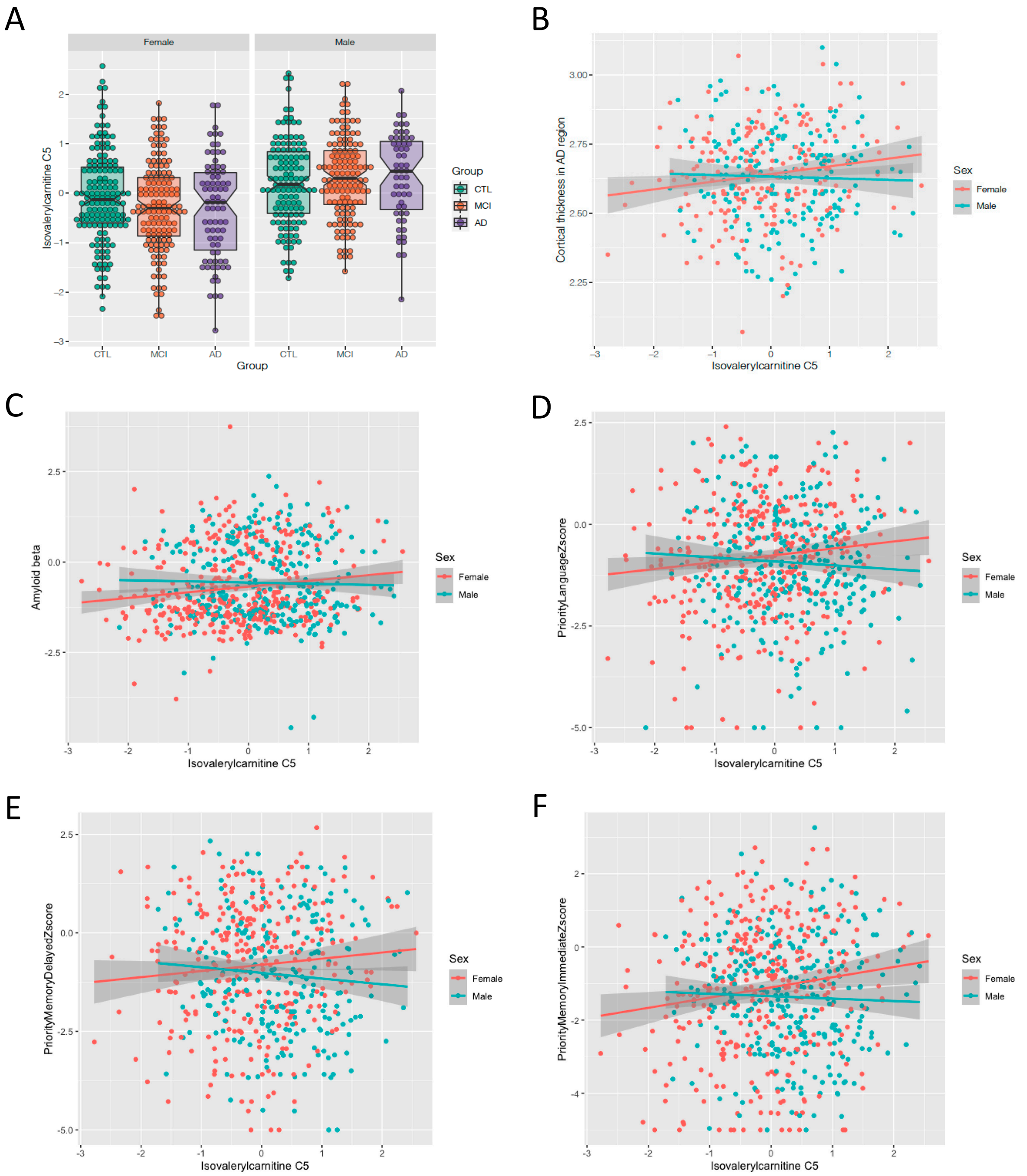

Figure S2. Isovalerylcarnitine C5 showed significant association in the female group with A) diagnosis (AD/CTL) (β = -0.46, 95% CI = -0.82 to -0.11, P = 1.1e-02); B) cortical thickness in AD region (β = 0.031, 95% CI = 0.0085 to 0.053, P = 7.3e-03); C) amyloid-beta (β = 0.17, 95% CI = 0.065 to 0.27, P = 1.3e-03); D) language z-score (β = 0.18, 95% CI = 0.043 to 0.33, P = 1.1e-02); E) memory delay z-score (β = 0.19, 95% CI = 0.0052 to 0.38, P = 4.5e-02)and F) memory immediate z-score (β = 0.29, 95% CI = 0.078 to 0.50, P = 7.5e-03).

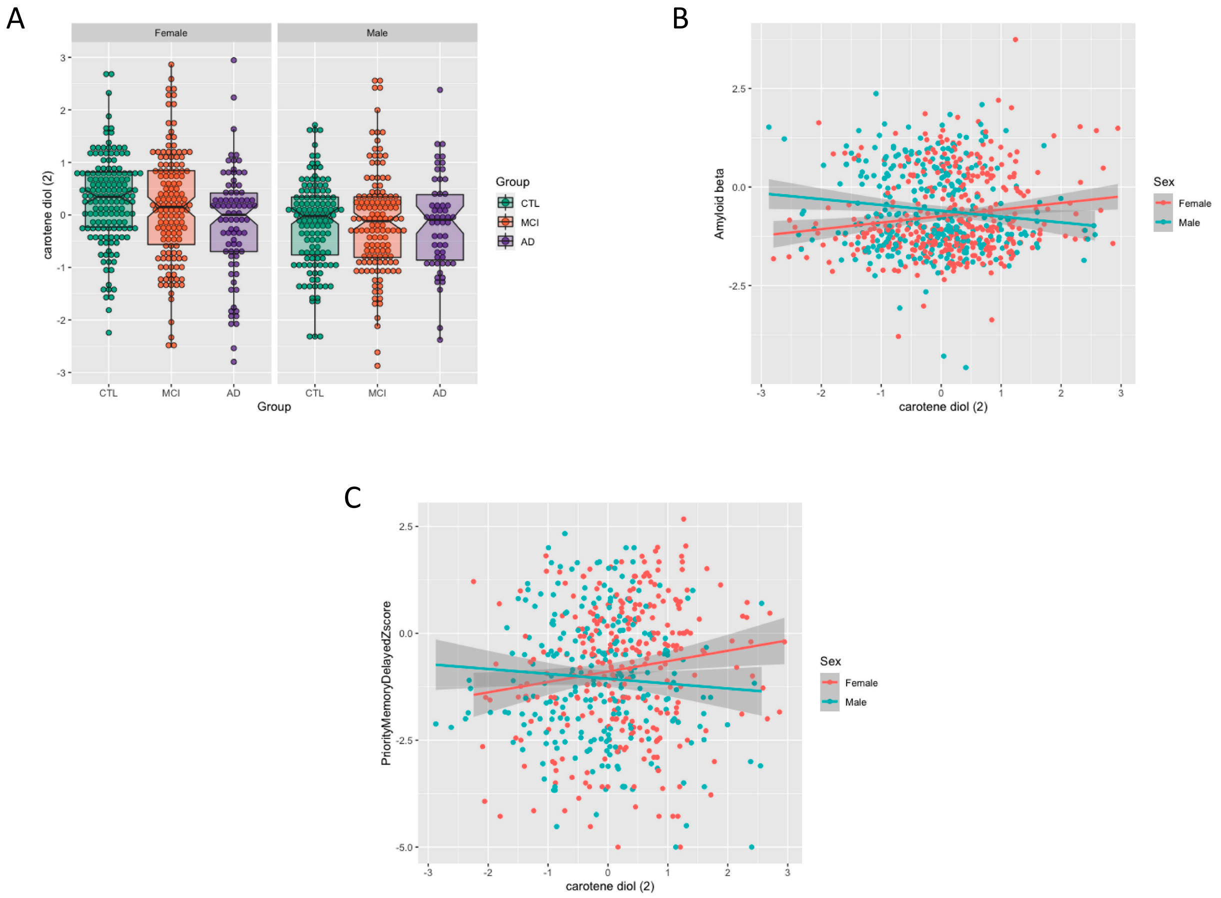

Figure S3. Carotene diol (2) showed significant association in the female group with A) diagnosis (AD/CTL) (β = -0.56, 95% CI = -0.93 to -0.22, P = 1.94e-03); B) amyloid-beta (β = 0.13, 95% CI = 0.033 to 0.23, P = 9.1e-03) and C) memory delay z-score (β = 0.022, 95% CI = 0.036 to 0.40, P = 1.9e-02).

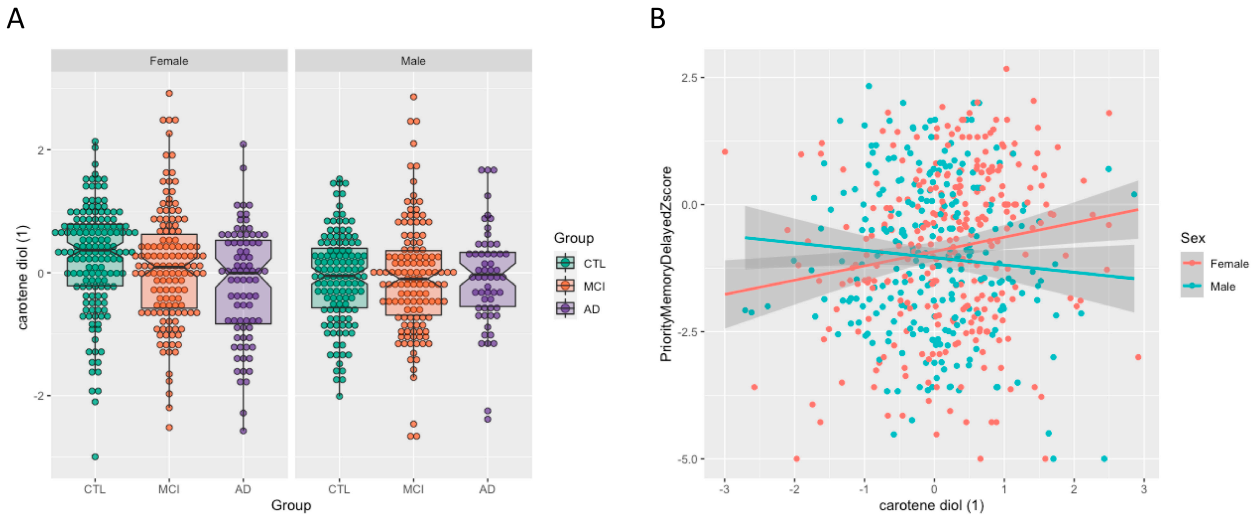

Figure S4. Carotene diol (1) showed significant association in the female group with A) diagnosis (AD/CTL) (β = -0.58, 95% CI = -0.96 to -0.22, P = 1.95e-03) and B) memory delay z-score (β = 0.21, 95% CI = 0.021 to 0.40, P = 3.0e-02).

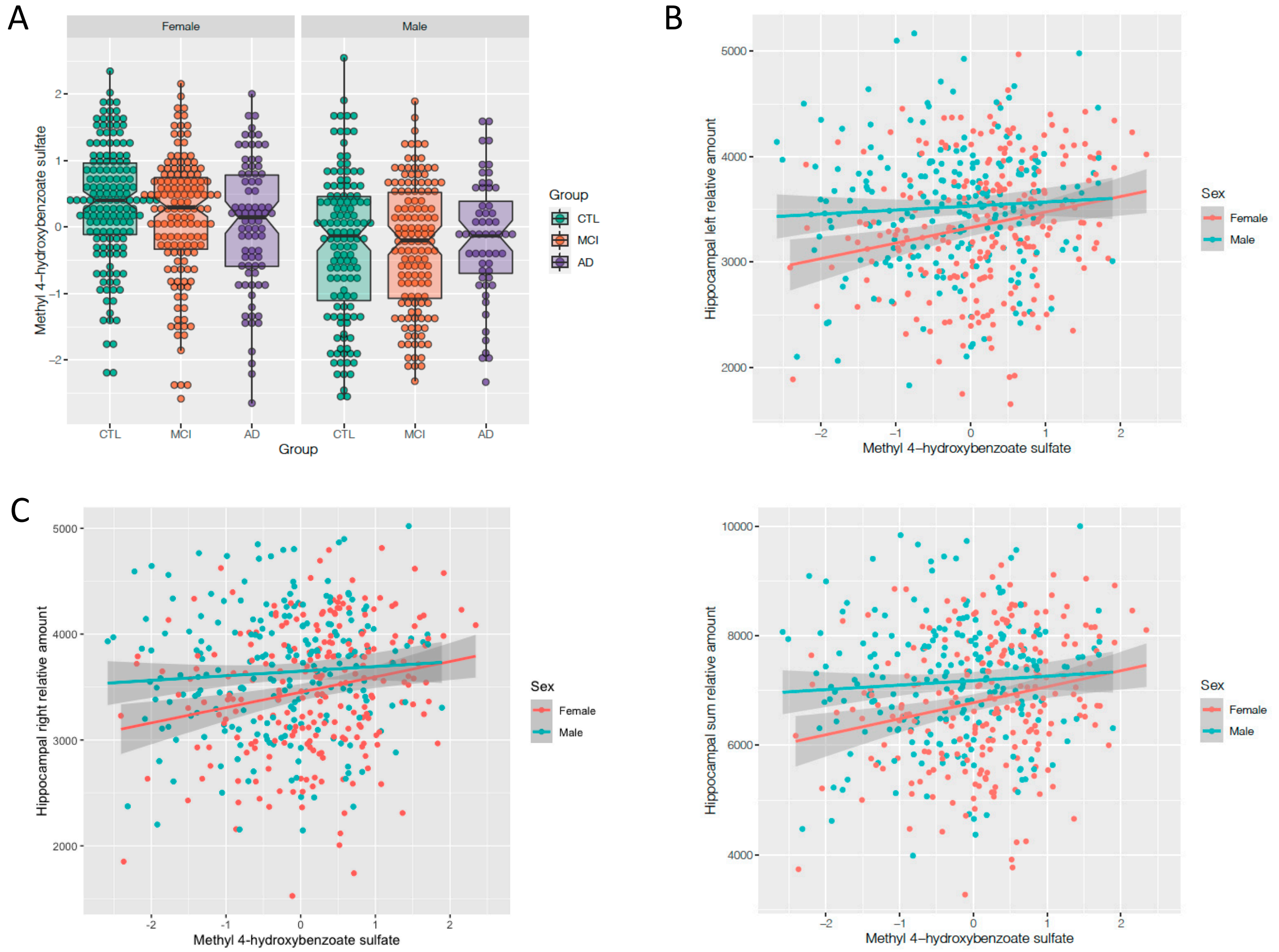

Figure S5. Methyl 4-hydroxybenzoate sulfate showed significant association in the female group with A) diagnosis (AD/CTL) (β = -0.41, 95% CI = -0.76 to -0.075, P = 1.8e-02); B) hippocampal left relative amount (β = 131.99, 95% CI = 51.86 to 212.13, P = 1.4e-03); C) hippocampal right relative amount (β = 123.74, 95% CI = 45.80 to 201.69, P = 2.1e-03) and D) hippocampal total relative amount (β = 255.73, 95% CI = 104.46 to 407.00, P = 1.1e-03).

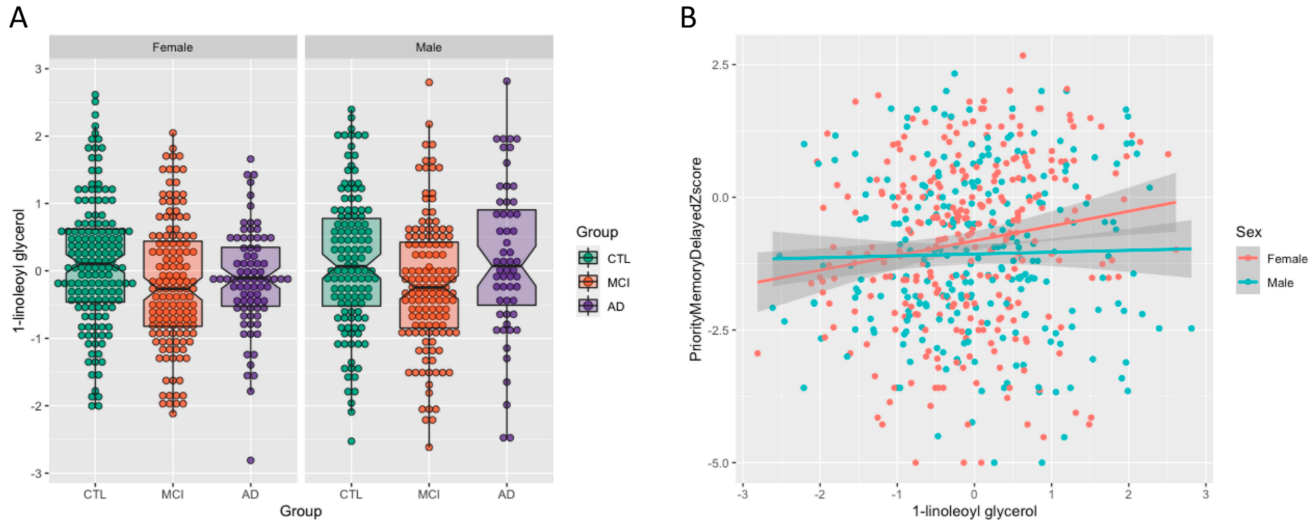

Figure S6. 1-linoleoyl glycerol showed significant association in the female group with A) diagnosis (AD/CTL) (β = -0.57, 95% CI = -0.98 to -0.18, P = 4.7e-03) and B) memory delay z-score (β = 0.27, 95% CI = 0.09 to 0.46, P = 3.7e-03).

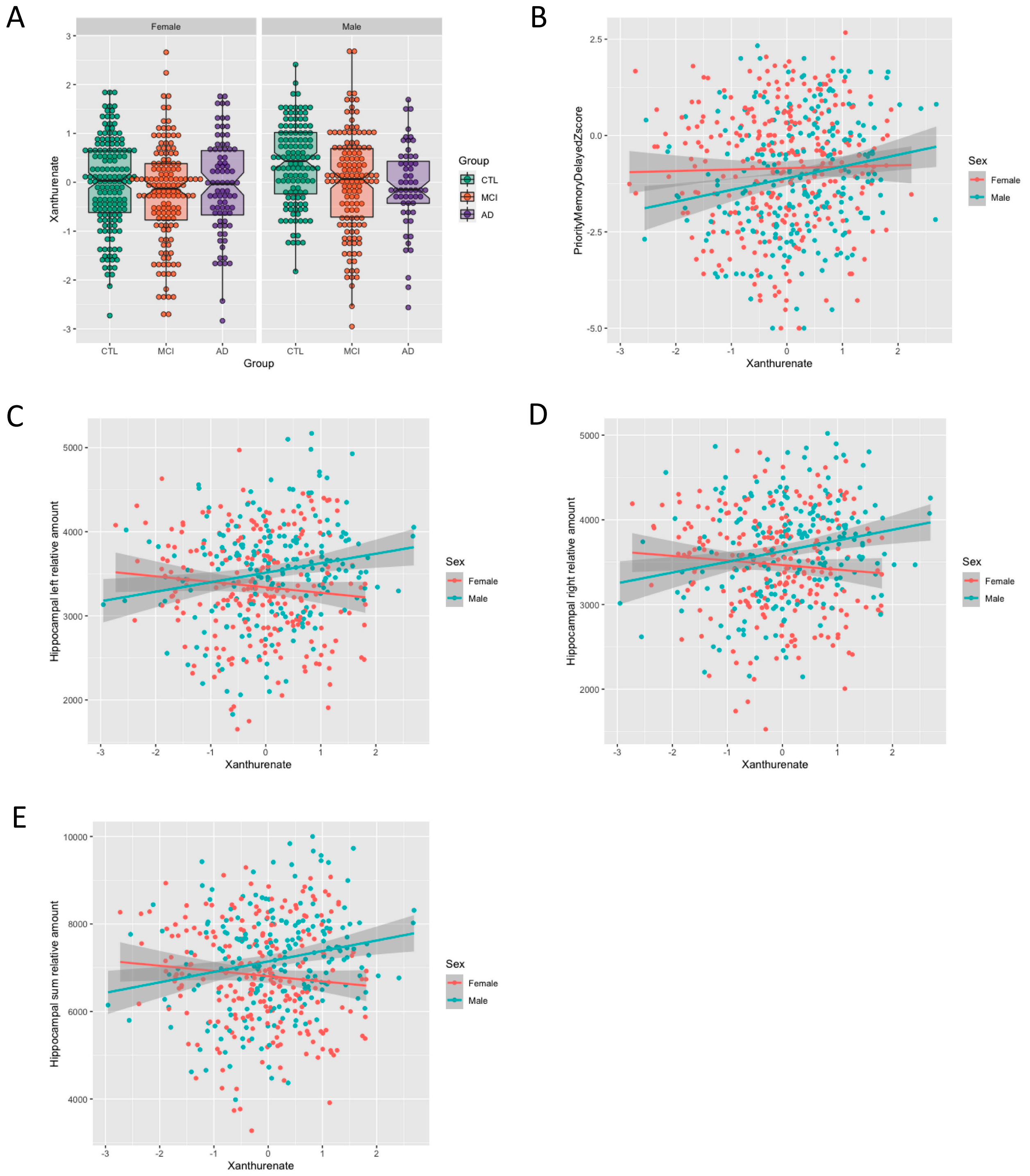

Figure S7. Xanthurenate showed significant association in the male group with A) diagnosis (AD/CTL) (β = -0.73, 95% CI = -1.18 to -0.31, P = 1.02e-03); B) amyloid-beta (β = 0.14, 95% CI = 0.026 to 0.25, P = 1.62e-02); C) hippocampal left relative amount (β = 83.28, 95% CI = 8.83 to 157.72, P = 2.9e-02); D) hippocampal right relative amount (β = 98.28, 95% CI = 25.45 to 171.11, P = 8.8e-03) and E) hippocampal total relative amount (β = 181.57, 95% CI = 41.18 to 321.95, P = 1.2e-02).

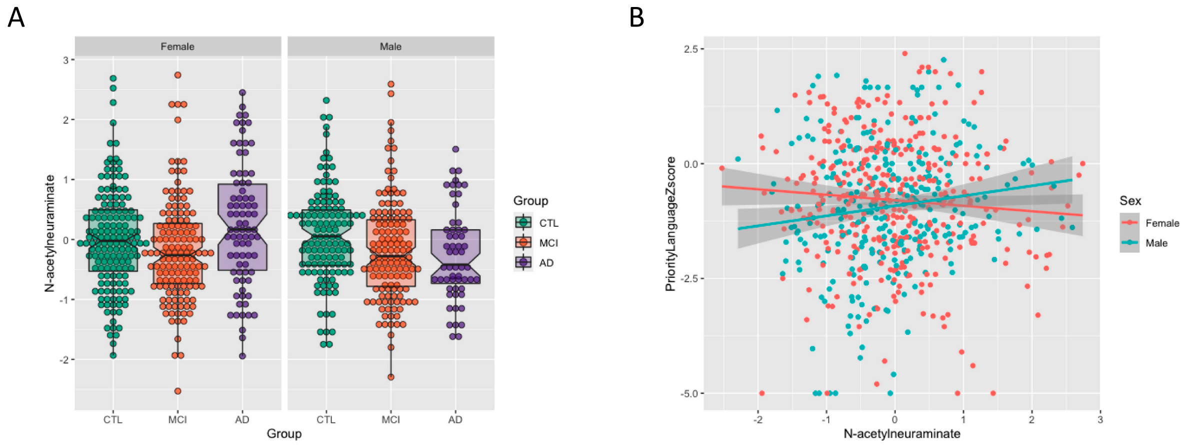

Figure S8. N-acetylneuraminate showed significant association in the male group with A) diagnosis (AD/CTL) (β = -0.64, 95% CI = -1.13 to -0.18, P = 8.03e-03) and B) language z-score (β = 0.23, 95% CI = 0.046 to 0.42, P = 1.5e-02).

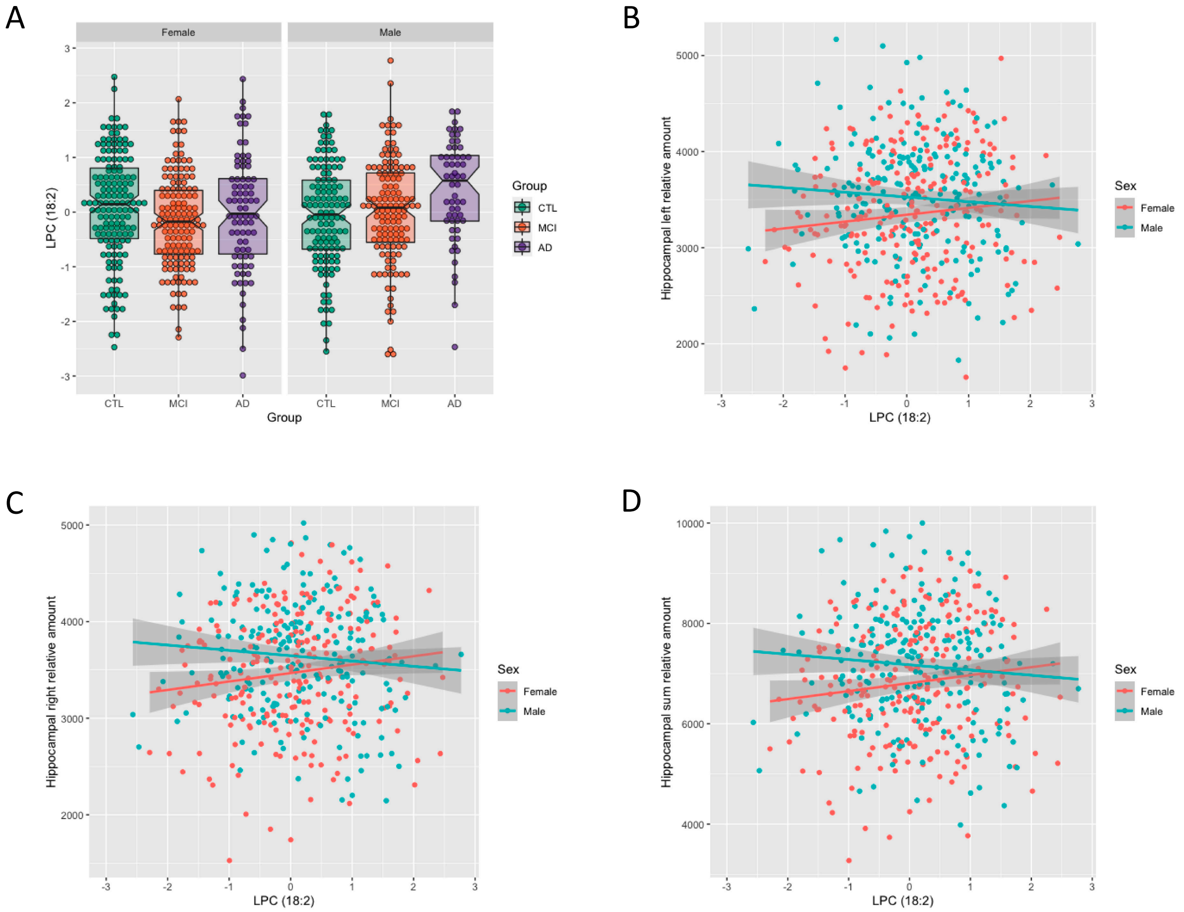

Figure S9. LPC (18:2) showed significant association in the male group with A) diagnosis (AD/CTL) (β = 0.69, 95% CI = 0.29 to 1.13, P = 1.1e-03), B) hippocampal left relative amount (β = -91.03, 95% CI = -170.63 to -11.43, P = 2.6e-02); C) hippocampal right relative amount (β = -98.73, 95% CI = -176.98 to -20.49, P = 1.5e-02) and D) hippocampal total relative amount (β = -189.75, 95% CI = -340.19 to -39.30, P = 1.42e-02).

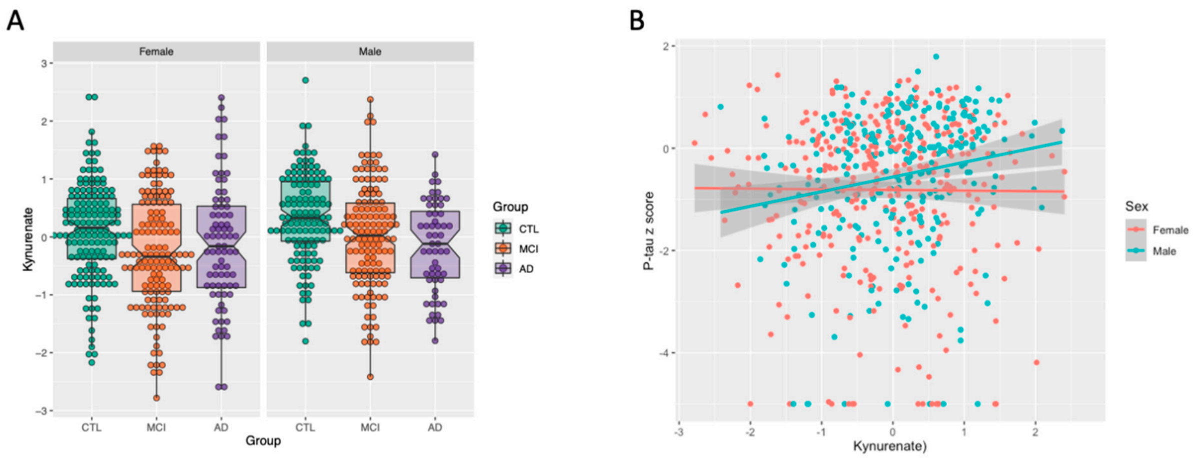

Figure S10. Kynurenate showed significant association in the male group with A) diagnosis (AD/CTL) (β = -1.04, 95% CI = -1.58 to -0.54, P = 7.6e-05) and B) p-tau z-score (β = 0.29, 95% CI = 0.11 to 0.47, P = 1.77e-03).

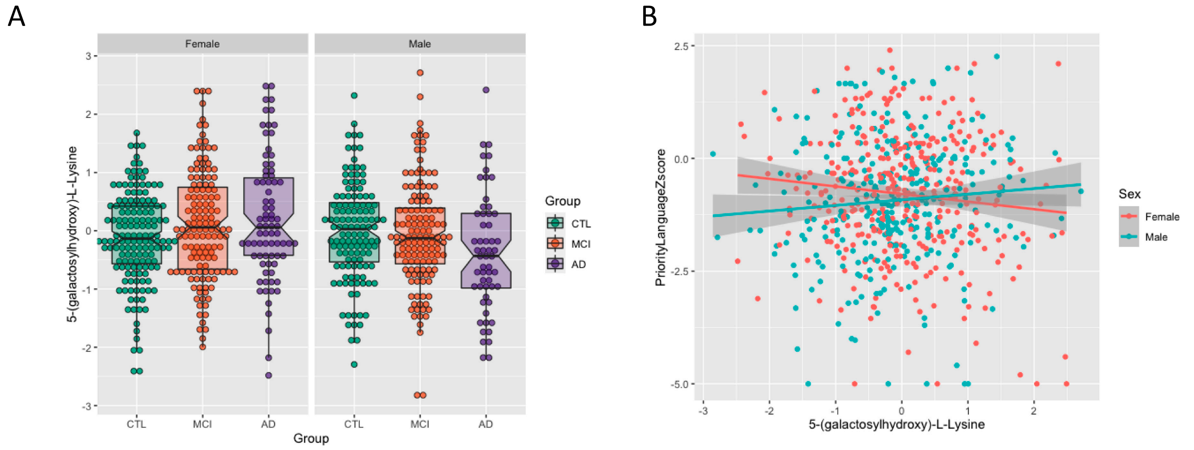

Figure S11. 5-(galactosylhydroxy)-L-Lysine showed significant association in the male group with A) diagnosis (AD/CTL) (β = -0.68, 95% CI = -1.12 to -0.26, P = 2.0e-03) and B) language z-score (β = 0.18, 95% CI = 0.0062 to 0.35, P = 4.3e-02).

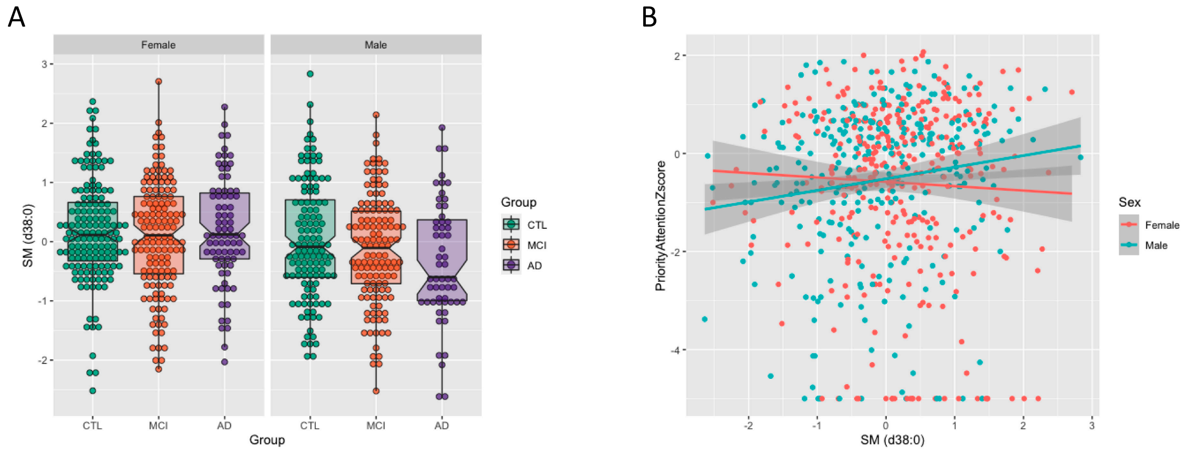

Figure S12. SM (d38:0) showed significant association in the male group with A) diagnosis (AD/CTL) (β = -0.47, 95% CI = -0.84 to -0.11, P = 1.2e-02) and B) attention z-score (β = 0.20, 95% CI = 0.016 to 0.39, P = 3.4e-02).

A

	Reference + VMA + Tryptophan betaine		Total	
Reference	<36.8%	>=36.8%		
event				NRI $_{event}$
<36.8%	7	6	13	=(6-3)/75
>=36.8%	3	59	62	=0.04
New total	10	65	75	
Non-event				NRI $_{non-event}$
<36.8%	103	4	107	=(9-4)/129
>=36.8%	9	13	22	=0.0388
New total	112	17	129	

NRI = 0.0788

B

	Reference + Kynurenate		Total	
Reference	<34.7%	>=34.7%		
event				NRI $_{event}$
<34.7%	9	0	9	=(0-0)/52
>=34.7%	0	43	43	=0
New total	9	43	52	
Non-event				NRI $_{non-event}$
<34.7%	79	3	82	=(3-3)/98
>=34.7%	3	13	16	=0
New total	82	16	98	

NRI = 0

C

	Reference + VMA + Tryptophan betaine		Total	
Reference	<35.9%	>=35.9%		
event				NRI $_{event}$
<35.9%	13	8	21	=(8-2)/127
>=35.9%	2	104	106	=0.0472
New total	15	112	127	
Non-event				NRI $_{non-event}$
<35.9%	183	8	191	=(8-6)/227
>=35.9%	9	27	36	=0.0044
New total	192	35	227	

NRI = 0.0516

D

	Reference + Kynurenate		Total	
Reference	<35.9%	>=35.9%		
event				NRI $_{event}$
<35.9%	21	0	21	=(0-1)/127
>=35.9%	1	105	106	=-0.0079
New total	22	105	127	
Non-event				NRI $_{non-event}$
<35.9%	188	3	191	=(3-3)/227
>=35.9%	3	33	36	=0
New total	191	36	227	

NRI = -0.0079

Figure S13. Net reclassification index table for sex-specific biomarkers. CSF biomarkers and covariates were used as baseline predictors in the reference models. The event, non-event, and total NRI were calculated for A) vanillylmandelate (VMA) and tryptophan betaine in the female sub-dataset; B) kynurenate in the male sub-dataset; C) VMA and tryptophan betaine in the full cohort; D) kynurenate in the full cohort.

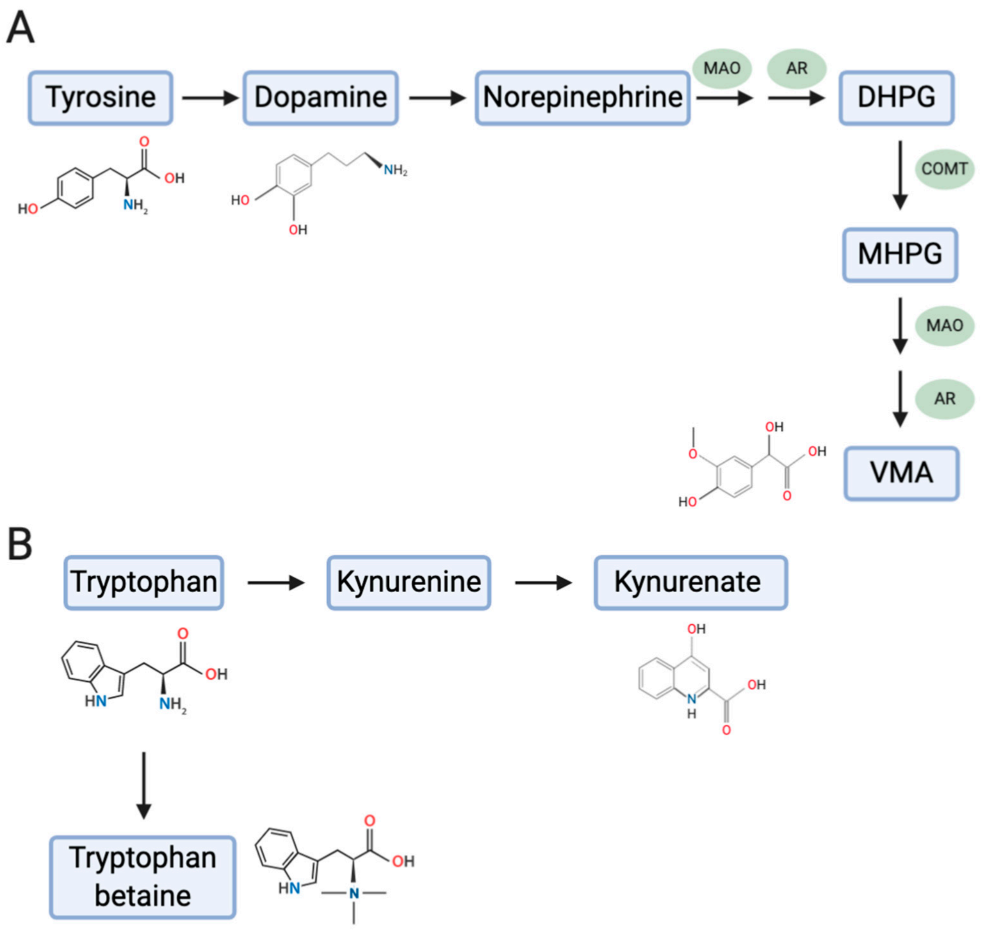

Figure S14. VMA, tryptophan betaine, and kynurenate were generated through tyrosine and tryptophan pathways. A) VMA is an end product of catecholamine metabolism in the tyrosine pathway metabolized by COMT, AR and MAO; B) kynurenate is produced from tryptophan in kynurenine pathway, while tryptophan betaine is mainly generated in plants and through gut microbiota.

VMA, vanillylmandelate; MAO, monoamine oxidase; COMT, catechol-O-methyl transferase; AR, aldehyde reductase; KAT, kynurenine aminotransferase. This figure is created with BioRender.com.

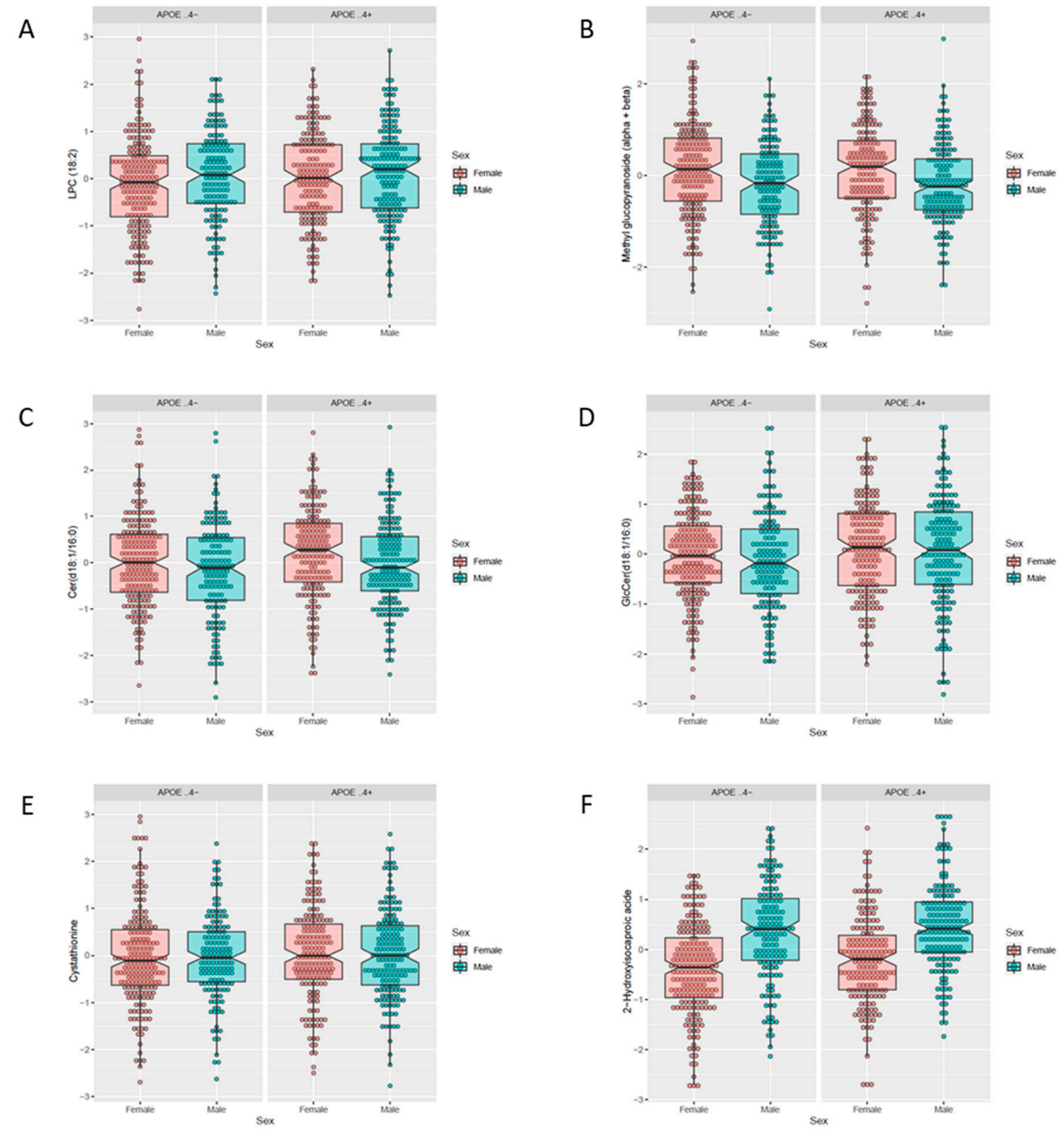

Figure S15. A) LPC (18:2) and B) methyl glucopyranoside (alpha + beta) showed association with AD at nominal level in both male and *APOE* ε4 carrier strata; C) Cer(d18:1/16:0) and D) GlcCer(d18:1/16:0) showed association with visuoconstruction at nominal level in both male and *APOE* ε4 carrier strata; E) cystathionine showed association with attention at nominal level in both male and *APOE* ε4 carrier strata; F) 2-Hydroxyisocaproic acid showed association with immediate memory at nominal level in both female and *APOE* ε4 carrier strata.
